# O Papel da Ecocardiografia sob Estresse na Detecção Precoce de Disfunção Diastólica em Pacientes com Doença Pulmonar Obstrutiva Crônica Não Grave

**DOI:** 10.36660/abc.20190623

**Published:** 2021-02-19

**Authors:** Zheyna Cherneva, Radostina Cherneva

**Affiliations:** 1 Medical InstituteMinistry of Internal AffairsSofiaBulgáriaMedical Institute of the Ministry of Internal Affairs, Sofia – Bulgária; 2 Saint Sophia University Hospital of Pulmonary DiseasesSofiaBulgáriaSaint Sophia University Hospital of Pulmonary Diseases, Sofia – Bulgária

**Keywords:** Ecocardiografia sob Estresse/métodos, Insuficiência Cardíaca Diastólica, Volume Sistólico, Doença Pulmonar Obstrutiva Crônica, Testes de Função Respiratória

## Abstract

**Fundamento:**

A dispneia por esforço é uma queixa comum de pacientes com insuficiência cardíaca com fração de ejeção preservada (ICFEP) e doença pulmonar obstrutiva crônica (DPOC). A ICFEP é comum na DPOC e é um fator de risco independente para a progressão e exacerbação da doença. A detecção precoce, portanto, tem grande relevância clínica.

**Objetivos:**

O objetivo deste estudo foi detectar a frequência de ICFEP mascarada em pacientes com DPOC não grave com dispneia aos esforços, sem doença cardiovascular manifesta, e analisar a correlação entre ICFEP mascarada e os parâmetros do teste cardiopulmonar de exercício (TCPE).

**Métodos:**

Aplicamos o TCPE em 104 pacientes com DPOC não grave com dispneia aos esforços, sem doença cardiovascular evidente. A ecocardiografia foi realizada antes e no pico do TCPE. Os valores de corte para disfunção diastólica ventricular esquerda e direita induzida por estresse (DDVE/DDVD) foram E/e’ >15; E/e’ >6, respectivamente. A análise de correlação foi feita entre os parâmetros do TCPE e o estresse E/d’. Valor de p<0,05 foi considerado significativo.

**Resultados:**

64% dos pacientes tinham DDVE induzida por estresse; 78% tinham DDVD induzida por estresse. Ambos os grupos com estresse DDVE e DDVD obtiveram carga menor, V’O_2_ e pulso de O_2 _mais baixos, além de apresentarem redução na eficiência ventilatória (maiores inclinações de VE/VCO_2_). Nenhum dos parâmetros do TCPE foram correlacionados com E/e’ DDVE/DDVD induzida por estresse.

**Conclusão:**

Há uma alta prevalência de disfunção diastólica induzida por estresse em pacientes com DPOC não grave com dispneia aos esforços, sem doença cardiovascular evidente. Nenhum dos parâmetros do TCPE se correlaciona com E/e’ induzida por estresse. Isso demanda a realização de Ecocardiografia sob estresse por exercício (EES) e TCPE para detecção precoce e manejo adequado da ICFEP mascarada nesta população. (Arq Bras Cardiol. 2021; 116(2):259-265)

## Introdução

As anormalidades cardiovasculares são comuns na doença pulmonar obstrutiva crônica (DPOC).^1,2^A rigidez arterial está presente mesmo em pacientes com DPOC leve e sem doenças cardiovasculares. Trata-se de um fator de risco cardiovascular independente que contribui para o desenvolvimento de disfunção diastólica.^3^ Dispneia e intolerância ao exercício são sintomas comuns da DPOC e disfunção diastólica.^4^ Estudos recentes com grandes coortes de pacientes identificaram um fenótipo cardiovascular em pacientes com DPOC que apresentam curso clínico e prognóstico diferentes.^5^ O diagnóstico e o manejo precoces são, portanto, muito importantes do ponto de vista clínico.

O teste cardiopulmonar de exercício (TCPE) pode distinguir dispneia cardíaca e respiratória ou diminuição da atividade física.^6-9^A combinação de ecocardiografia sob estresse por exercício (EES) e TCPE é uma abordagem confiável para identificar pacientes com insuficiência cardíaca mascarada pela fração de ejeção preservada (ICFEP). Além disso, os resultados das medições invasivas são comparáveis aos dados obtidos em estudos não invasivos durante o EES.^10^

Os objetivos de nosso estudo foram: 1) detectar a frequência de disfunção diastólica subclínica do ventrículo esquerdo (VE) e do ventrículo direito (VD) em pacientes com DPOC não grave sem doença cardiovascular; 2) estabelecer uma correlação entre exercício cardiopulmonar e os parâmetros ecocardiográficos para disfunção diastólica (E/e’).

## Materiais e métodos

### Pacientes e protocolo de estudo

Este foi um estudo retrospectivo realizado com 224 pacientes ambulatoriais clinicamente estáveis com diagnóstico de DPOC no Hospital Universitário de Doenças Respiratórias “St. Sophia”, Sofia. Apenas 163 deles atenderam aos critérios de inclusão para DPOC não grave: volume expiratório forçado no primeiro segundo maior que 50% (VEF_1_>50%). Todos os indivíduos apresentavam dispneia aos esforços, mas um total de 104 pacientes (64 homens, 40 mulheres; idade média de 62,9±7,5 anos) foram considerados elegíveis, assumindo-se os critérios de exclusão. O período de recrutamento foi entre maio de 2017 e abril de 2018, e foi aprovado pelo Comitê de Ética local (protocolo 5/12.03.2018). Todos os participantes assinaram o termo de consentimento livre e esclarecido antes do início do estudo.

Foram considerados os seguintes critérios de exclusão: 1) fração de ejeção do ventrículo esquerdo (FEVE) <50%; 2) disfunção diastólica do ventrículo esquerdo em repouso superior ao primeiro grau; 3) achados ecocardiográficos sugerindo hipertensão pulmonar (pressão arterial pulmonar sistólica >36 mmHg, regurgitação tricúspide (RT), velocidade máxima do jato >2,8 m/s; 4) cardiopatia valvar; 5) cardiomiopatia documentada; 6) hipertensão grave não controlada (pressão arterial sistólica >180 mmHg e pressão arterial diastólica >90 mmHg); 7) fibrilação atrial ou arritmia ventricular maligna; 8) doença isquêmica do coração; 9) anemia; 10) diabetes mellitus; 11) câncer; 12) doença renal crônica (DRC); 13) cirurgia torácica ou abdominal recente; 14) exacerbação recente (nos últimos três meses); 15) mudança recente (nos últimos três meses) na terapia médica.

## Procedimentos

### Teste de função pulmonar

Todos os indivíduos foram submetidos a um exame clínico preliminar, incluindo radiografia de tórax, espirometria, eletrocardiografia e ecocardiografia. Os pacientes elegíveis para o estudo realizaram espirometria e teste ergométrico em Vyntus, teste cardiopulmonar de exercício (Carefusion, Alemanha), de acordo com as diretrizes da European Respiratory Society (ERS).^11^ Apenas pacientes com obstrução leve a moderada das vias aéreas (VEF_1_> 50%) foram selecionados.

### Protocolo de teste de estresse – teste cardiopulmonar de exercício (TCPE)

Um protocolo em rampa contínua foi aplicado de acordo com diretrizes.^6^ Após dois minutos de pedalada sem carga (fase de repouso 0W), seguiu-se uma fase de aquecimento de três minutos (20W). A fase de teste incluiu incrementos de carga de 20W/2min. Os pacientes foram orientados a pedalar a 60 rotações por minuto.

Os gases expiratórios foram coletados respiração a respiração. O pico de VO_2_ foi expresso como o maior valor médio de 30 segundos, obtido na última etapa do teste de esforço. Os valores de pico de VO_2_ são expressos como -O_2_ ml/kg/min. A eficiência ventilatória (EV/VCO_2_) foi medida pelo método V-slope. O pico da razão de troca respiratória (pRTR) foi o maior valor médio de 30 segundos no último estágio do teste. RTR >1,10 ao final do teste EES-TCPE foi considerado como esforço máximo.

## Métodos ecocardiográficos

Foram realizados ecocardiografia modo M, bidimensional com Doppler.^12,13^ Incidências apicais de quatro câmaras foram usadas para medir os volumes das câmaras com base na regra modificada de Simpson e a fração de ejeção do VE foi considerada preservada se >50%. A análise da ecocardiografia Doppler tecidual foi realizada na dimensão septo-lateral do anel mitral e na dimensão lateral do anel tricúspide para avaliar as ondas sistólica (S) e diastólica miocárdica (E’, A’) do VE e VD. O valor E’ foi usado como média das medições mediais e laterais. O pico da razão E/e’ >15 foi considerado um marcador para disfunção diastólica ventricular esquerda induzida por estresse.

A função sistólica do ventrículo direito foi avaliada por meio da excursão sistólica do plano do anel tricúspide (TAPSE) e da velocidade de pico S do Doppler tecidual. A espessura da parede do ventrículo direito (EPVD) foi medida a partir do corte subcostal no eixo longo na extremidade do folheto tricúspide anterior ao final da diástole. A pressão pulmonar foi calculada diretamente pela amostra da insuficiência tricúspide e indiretamente pelo tempo de aceleração (TA) do fluxo pulmonar. O índice de volume do átrio direito (IVAD) foi medido com o volume sistólico final do ventrículo direito pela regra modificada de Simpson. A disfunção diastólica do VD induzida por estresse foi considerada se a razão E/e’ induzida por estresse fosse >6. Todos os parâmetros foram medidos no final da expiração e três vezes durante diferentes ciclos cardíacos.^14^

### Análise estatística

Os dados demográficos e clínicos foram apresentados em estatística descritiva. O teste de Kolmogorov-Smirnov foi usado para explorar a normalidade da distribuição. As variáveis contínuas em cada grupo foram expressas como mediana e intervalo interquartil quando os dados não foram normalmente distribuídos, e como média ± desvio-padrão (DP) se a distribuição foi normal. Variáveis categóricas foram apresentadas em proporções. Os dados foram comparados entre pacientes com e sem DDVE, bem como entre pacientes com e sem DDVD. O teste t de Student não pareado foi usado para analisar variáveis contínuas normalmente distribuídas. O teste de Mann-Whitney-U foi usado em outros casos. Variáveis categóricas foram comparadas pelo teste de χ^2^ ou exato de Fisher. A correlação de Spearman foi usada para avaliar a associação entre os parâmetros TCPE e a razão E/e’ induzida por estresse nos ventrículos esquerdo e direito.

Em todos os casos, um valor de p inferior a 0,05 foi considerado significativo, conforme determinado pelas estatísticas do software SPSS® 13.0 (SPSS, Inc, Chicago, Ill).

## Resultados

No grupo DPOC, 30% (32/104) dos pacientes apresentaram DDVE grau I em repouso; 14% (15/104) tinham DDVD grau I em repouso e apenas 3% (4/104) tinham tanto DDVE quanto DDVD em repouso. Após o TCPE, a ecocardiografia sob estresse estabeleceu que 64% (67/104) dos indivíduos tinham DDVE induzida por estresse e 78% (82/104) tinham DDVD induzida por estresse. Todos os pacientes com DDVE induzida por estresse também tinham DDVD induzida por estresse. Os dados demográficos e clínicos dos pacientes estão listados na Tabela 1. Os parâmetros ecocardiográficos dos pacientes estão apresentados na Tabela 2. Exceto para IVAD, EPVD, TA e pressão arterial pulmonar sistólica após carga, nenhuma outra diferença significativa foi encontrada entre os pacientes com e sem DDVE (Tabelas 1 e 2). Os resultados para pacientes com e sem DDVD foram semelhantes (Tabelas 1 e 2).

**Tabela 1:** – Características antropométricas e cardiopulmonares de pacientes com e sem DDVE e DDVD

	Pacientes sem DDVE por estresse (37)	Pacientes com DDVE por estresse (67)	Pacientes sem DDVD por estresse (22)	Pacientes com DDVD por estresse (82)
**Dados demográficos**				
Idade, ano	60,44 ± 7,72	64,16 ± 6,97*	6,95±7,36	63,74±7,60*
Masculino:feminino, n	21:16	44:23^ǂ^	14:8	50:32^ǂ^
Pacote, anos	27,21 (23,87-31,76)	33,79 (30,51± 37,87)^†^	26,52 (23,46-30,43)	32,11(28,82-36,13)^ǂ^
Índice de massa corpórea, kg/m^2^	27,00 (24,75-31,00)	27,96 (22,75-30,75)^†^	28,00 (25,25-30,5)	26,52 (22,72-30,61)^†^
**Função respiratória**				
CVF, l/min	2,06 (1,76-3,09)	2,34 (1,77-3,09)^†^	2,05 (2,11-3,73)	2,21 (1,71-2,93)^†^
FEV_1_, l/min	1,31 (0,94-1,53)	1,36 (1,14-1,75)^†^	1,60 (1,15-2,42)	1,52 (1,14-1,75)^†^
FEV_1_/CVF %	60,5 (46,91-67,47)	53,30 (45,76-66,55)^†^	65,50 (54,81-68,82)	62,59 (46,57-66,79)^†^
**Equilíbrio ácido-base**				
pO_2_, mmHg	68,60(63,4-71,8)	71,35 (64,7-74)^†^	67,20 (63,56-71,68)	70,6 (63,2-74)^†^
pCO_2_, mmHg	32,30 (30,1-35,37)	37,65 (32,5-40)^†^	34,73 (31,27-39,21)	35,7 (32,5-40)^†^
Saturação, %	94,9 (94,4-95,25)	95,00 (94,02-95,67)^†^	94,75 (92,67-95,0)	95,00 (93,9-95,5)^†^
**Parâmetros TCPE**				
Pico de carga, W	82,75 (69,8-89,1)	▪ 76,05 (68,4-92,1)^†^	86,66 (78,65-94,76)	▪▪73,08 (68,93-83,16)^†^
Pico de VM, l/min	40 (34-52,5)	38,50 (32-48)^†^	41,1 (32,12-48,17)	39,07 (31,89-48,32)^†^
Pico de V’O_2_, ml/min/kg	14,30(12,6-16,15)	13,90 (12,67-15,7)^†^	14,30 (12,6-16,15)	13,40(15,77-12,55)^†^
RTR	1,06 (0,98-1,19)	1,09 (1,00-1,28)^†^	1,05 (0,98-1,18)	1,08 (1,01-1,19)^† ^
Pico de pulso de O_2_ ml/min/kg	9,80 (9,5-12,2)	▪7,90 (6,15-9,32)^†^	9,51 (9,02-13,1)	▪▪7,92(6,27-9,84)^†^
Pico de inclinação VM/VCO_2_	34,08 (33,98-36,72)	▪36,93 (34,19-38,74)^†^	34,11 (33,78-36,89)	▪▪36,98 (34,26-38,91)^†^

*Teste t não pareado; †Teste U de Mann-Whitney; Teste ǂchi-quadrado; DDVE: disfunção diastólica do ventrículo esquerdo; DDVD: disfunção diastólica do ventrículo direito; CVF: capacidade vital forçada; Pulso de O^*2*^: pulso de oxigênio; VM: ventilação minuto; RTR: razão de troca respiratória; V’O^*2*^: consumo de oxigênio; TCPE: parâmetros do teste cardiopulmonar de exercício. Inclinação VM/VCO^*2*^: eficiência ventilatória; ▪p<0,05 entre pacientes com e sem DDVE; ▪▪p<0,05 entre pacientes com e sem DDVD.

**Tabela 2:** – Parâmetros ecocardiográficos de pacientes com e sem DDVE e DDVD

	Pacientes sem DDVE por estresse (37)	Pacientes com DDVE por estresse (67)	Pacientes sem DDVD por estresse (22)	Pacientes com DDVD por estresse (82)
**Parâmetros estruturais do VE**				
FEVE, %, Simpson	63,50(60-66)	60,00(57-65)*	65,00(60-66)	61,00 (67-65)*
Septo, mm	12,00(11-13)	12,00(11-13) *	12,00 (11-12,75)	12,00 (11-13)*
PW, mm	12,00(11,75-12)	12,00(11-13) *	12,00 (11,25-12,75)	12,00 (11-13)*
**Parâmetros funcionais do VE em repouso**				
Razão E/A	0,79(0,75-0,85)	0,85 (0,76-1,20)*	0,78 (0,76-0,83)	0,84 (0,75-1,21,)*
Razão média E/e’	6,66 (6,25-8,33)	6,97 (5,76-8,15)*	6,96 (6,27-8,33)	6,66 (5,63-8,1)*
**Parâmetros funcionais do VE após o teste de esforço**				
Razão E/A	1,25(0,8-1,5)	ǂ1,73 (1,55-2,00)*	1,22 (0,88-1,37)	ǂǂ1,71 (1,5-2,00)*
Razão média E/e’	8,07 (6,7-9,6)	ǂ17,33 (15,71-8,46)*	8,12 (7,25-10)	ǂǂ17,14 (14,66-18,39)*
**Parâmetros estruturais do VD**				
IVAD, ml/m^2^	17,57 (16,07-19,97)	ǂ22,66 (21,31-24,13)*	16,55 (15,81-17,54)	ǂǂ22,27 (20,65-23,85)*
RWT, mm	5,00 (4,5-6,5)	ǂ6,50 (6-7)*	5,00 (4,12-5,00)	ǂǂ6,50 (6,00-7,00)*
TAPSE, mm	23,00 (22,00-26,00)	22,00 (21,00-23,00)*	23,00 (21,25-26,00)	22,00 (21-23,5)*
**Parâmetros funcionais do VD em repouso**				
Razão E/A	0,83 (0,75-0,95)	0,69 (0,62-0,75)*	0,83 (0,76-1,16)	0,71 (0,66-0,83)*
Razão média E/e’	5,47 (4,56-5,69)	4,16(3,33-5,00)*	5,47 (4,56-5,69)	4,54(3,33-5,22)*
TA, msec	170 (163,75-180)	170(160-180)*	170 (165-180)	170(160-180)*
sPAP, mmHg	26,00 (25-28)	28,00 (25-30)*	25,00 (23-27)	28,00 (25-30)*
**Parâmetros funcionais do VD após o teste de esforço**				
Razão E/A	1,26 (1,09-1,48)	1,31(1,18-1,49)*	1,28 (1,14-1,5)	1,37 (1,22-1,52)*
Razão média E/e’	6,21 (5,38-7,89)	10,83 (9,04-13,23)*	6,92 (5,46-8,00)	ǂǂ11,25 (9,00-13,33)*
TA, msec	165(155-175)	ǂ105(95-110)*	162,5(155-170)	ǂǂ110(95-115)*
sPAP, mmHg	32,00(30-33,25)	ǂ38,00 (36-42)*	32,00 (30-33,75)	ǂǂ 38,00 (35-40)*

DDVE: disfunção diastólica do ventrículo esquerdo; DDVD: disfunção diastólica do ventrículo direito; FEVE: fração de ejeção do ventrículo esquerdo; IVAD: índice de volume do átrio direito; TAPSE: excursão sistólica do plano do anel tricúspide; ǂp <0,05 entre pacientes com e sem DDVE; PW: parede posterior; SPAP: pressão arterial pulmonar sistólica; EPVD: espessura da parede do ventrículo direito; TA: tempo de aceleração. ǂǂp <0,05 entre pacientes com e sem DDVD.

A capacidade de exercício foi reduzida em pacientes com DPOC com disfunção diastólica direita e esquerda induzida por estresse, em comparação com pacientes sem (Tabela 1). Os pacientes DPOC-DDVD/DDVE alcançaram carga mais baixa, VO_2_ e pulso de O_2_ mais baixos. Tiveram um desempenho com inclinações de VM/VCO_2_ significativamente maiores (Tabela 1). Nenhum dos parâmetros TCPE foi associado à razão E/e’ esquerda ou direita induzida por estresse (Tabela 3.)

**Tabela 3:** – Análise de correlação entre os parâmetros de teste de exercício respiratório e cardiopulmonar com a razão E/e’ induzida por estresse para ventrículo esquerdo/ventrículo direito, respectivamente

Parâmetros	DDVE		DDVD	
	Spearman rho	valor de p	Spearman rho	valor de p
Pico de carga, W	0,02	0,84	0,03	0,78
Pico de VM, l/min	0,02	0,85	0,12	0,28
Pico de VO_2_, ml/min/kg	0,12	0,56	0,03	0,73
RTR	0,06	0,74	0,12	0,27
Pico de pulso de O_2_ ml/min/kg	0,10	0,60	0,11	0,32
Pico de inclinação VM/VCO_2_	0,35	0,07	0,02	0,80
CVF, l/min	0,28	0,11	0,10	0,34
FEV_1_, l/min	0,01	0,95	0,04	0,71

Teste U de Mann-Whitney; † Abreviaturas: DDVE: disfunção diastólica do ventrículo esquerdo; DDVD: disfunção diastólica do ventrículo direito; CVF: capacidade vital forçada; RTR: razão de troca respiratória; ǂp<0,05 entre pacientes com e sem DDVE; VM/VCO_*2:*_ eficiência ventilatória; ǂǂp<0,05 entre pacientes com e sem DDVD

## Discussão

Nossos principais achados foram: 1) 64% dos pacientes com DPOC não grave e dispneia aos esforços que estão livres de doença cardiovascular clinicamente evidente têm DDVE induzida por estresse; 1) 78% do mesmo grupo de pacientes têm DDVD induzida por estresse; 3) nenhum dos parâmetros TCPE foi correlacionado com a razão E/e’ induzida por estresse no ventrículo esquerdo ou direito. Até onde sabemos, este é o primeiro estudo usando a combinação EES-TCPE em pacientes com DPOC não grave, dispneia aos esforços e livres de doenças cardiovasculares evidentes. Aumento da razão E/e’ induzida por estresse >15 do ventrículo esquerdo foi detectado em 64% deles; elevação induzida por estresse da razão E/e’ >6 no ventrículo direito foi encontrada em 78% dos casos. Não podemos comparar nossos dados com outros estudos de populações de pacientes com DPOC não grave porque a maioria deles relata a incidência de disfunção diastólica em repouso.^15-17^

Nedeljkovic et al. realizaram EES em uma população de 87 pacientes hipertensos com dispneia aos esforços e função ventricular esquerda normal. Reportaram, em 9,2% dos pacientes, razão por estresse E/e’ >15.^18^ Kaiser et al. também investigaram uma população geral de 87 pacientes com dispneia aos esforços e relataram disfunção diastólica em 9% deles.^19^

A maior prevalência de disfunção diastólica de estresse que descrevemos em pacientes com DPOC confirma que a própria DPOC é um fator de risco cardiovascular.^20,21^ Rigidez arterial é uma característica da DPOC, independentemente do impacto do tabagismo. O estresse da parede ventricular observado durante a respiração também é relatado como um mecanismo fisiopatológico independente para a remodelação do VE em pacientes com DPOC leve sem patologia cardiovascular evidente.^22^ Tanto a rigidez arterial quanto o estresse da parede ventricular causam fibrose ventricular difusa em pacientes com DPOC livres de doenças cardiovasculares.^23,24^

Em nosso estudo, pacientes com disfunção diastólica induzida por estresse (ambos DDVE e DDVD) atingem carga, VO_2_ e pulso de O_2_ mais baixos e apresentam desempenho em inclinações de VM/VCO_2_ significativamente maiores. Nenhum dos parâmetros TCPE, no entanto, se correlaciona com a razão de estresse E/e’ (nem no VE nem no VD). Esses achados são semelhantes aos relatados na população em geral. Nedeljkovic et al. detectaram menor carga, menor consumo de oxigênio e menor eficiência ventilatória em pacientes hipertensos com dispneia aos esforços e DDVE induzida por estresse.^18^ Kaiser et al. descreveram aumento da reserva de frequência cardíaca e redução do pulso de oxigênio em uma população geral de pacientes com dispneia aos esforços.^19^ Guazzi et al. também estabeleceram uma associação entre disfunção diastólica (razão E/e’) e consumo ao pico de oxigênio, eficiência ventilatória e recuperação da frequência cardíaca.^25^ No grupo de pacientes com patologia cardiovascular evidente e ecocardiografia normal em repouso de Guazzi, a eficiência ventilatória se correlacionou melhor com o pico da razão E/e’ >15. A vantagem clínica da relação VM/VCO_2_ como melhor preditor da razão por estresse E/e’ também foi confirmada nos pacientes com insuficiência cardíaca diastólica analisados por Nedeljkovic et al.^18^ Kaiser et al. não apoiam tais conclusões, enfatizando a importância do aumento da reserva da frequência cardíaca e da diminuição no pulso de oxigênio como preditores da razão por estresse E/e’ na população geral de pacientes com dispneia aos esforços e livres de doença cardiovascular evidente.^19^

Parece que os parâmetros TCPE podem ajudar no diagnóstico diferencial de dispneia na população em geral, bem como em pacientes com patologia cardiovascular diagnosticada.^6-9^ De acordo com nossos resultados, em pacientes com DPOC, esses não são parâmetros clínicos confiáveis que podem servir como preditores independentes de anormalidade cardiovascular e, portanto, não são aplicáveis no algoritmo de diagnóstico de disfunção diastólica mascarada.

Nossos achados corroboram a presença de comprometimento funcional em pacientes com DPOC não grave com dispneia aos esforços e livres de doença cardiovascular evidente. O Doppler tecidual durante o exercício mostra a complexa interação coração-pulmão e as alterações induzidas pelo esforço, que aumentam os comprometimentos funcionais cardíacos que podem não ser evidentes em repouso. Nossos achados apoiam as recomendações atuais de EES-TCPE como uma ferramenta para detecção precoce de ICFEP.^6^ Como nenhum dos parâmetros do teste de esforço cardiopulmonar provou ser preditivo de DDVE/DDVD induzida por estresse, a ecocardiografia de estresse tem grande relevância clínica para um diagnóstico preciso de patologia cardíaca e respiratória em pacientes com DPOC não graves e dispneia aos esforços.

### Limitações do estudo

O estudo teve as seguintes limitações: 1) tamanho de amostra relativamente pequeno; 2) falta de pletismografia corporal e medida da capacidade de difusão, que são informativos para uma avaliação adequada da dispneia; 3) pacientes com DPOC apresentam oscilações de pressão aumentada durante o ciclo respiratório, e a medição foi realizada ao final da expiração, o que pode influenciar os resultados; 4) as medições foram feitas no início do período de recuperação (aproximadamente dois minutos) após o exercício limitado por sintomas. A linha do tempo das mudanças das pressões pulmonar e intratorácica durante o breve intervalo entre o pico do exercício e sua medida no início da recuperação não é bem conhecida e poderia, portanto, ser subestimada.

## Conclusão

Há uma alta prevalência de disfunção diastólica induzida por estresse em pacientes com DPOC não grave com dispneia aos esforços, sem doença cardiovascular evidente. Nenhum dos parâmetros do TCPE se correlaciona com a razão E/e’ induzida por estresse. Portanto, a realização do EES-TCPE é necessária para a detecção precoce e o manejo adequado da ICFEP mascarada nesta população.
