# Supplementary material for: O Papel da Ecocardiografia sob Estresse na Detecção Precoce de Disfunção Diastólica em Pacientes com Doença Pulmonar Obstrutiva Crônica Não Grave
Source: Arq Bras Cardiol. 2021 Feb 19;116(2):259–65. [Article in Portuguese] doi: 10.36660/abc.20190623 (PMC7909987; doi:10.36660/abc.20190623)
Supplement: Translation

# Translation: The Role of Stress Echocardiography in the Early Detection of Diastolic Dysfunction in Non-Severe Chronic Obstructive Pulmonary Disease Patients

**Authors:** Zheyna Cherneva, Radostina Cherneva

**Affiliations:** 1 BulgáriaMedical Institute of the Ministry of Internal Affairs, Sofia - Bulgária; 2 BulgáriaSaint Sophia University Hospital of Pulmonary Diseases, Sofia - Bulgária

**Keywords:** Echocardiography,Stress/methods, Heart Failure,Diastolic, Stroke Volume, Pulmonary Disease,Chronic Obstructive, Respiratory Funstion Tests

## Abstract

**Background:**

Exertional dyspnea is a common complaint of patients with heart failure with preserved ejection fraction (HFpEF) and chronic obstructive pulmonary disease (COPD). HFpEF is common in COPD and is an independent risk factor for disease progression and exacerbation. Early detection, therefore, has great clinical relevance.

**Objectives:**

The aim of the study is to detect the frequency of masked HFpEF in non-severe COPD patients with exertional dyspnea, free of overt cardiovascular disease, and to analyze the correlation between masked HFpEF and the cardiopulmonary exercise testing (CPET) parameters.

**Methods:**

We applied the CPET in 104 non-severe COPD patients with exertional dyspnea, free of overt cardiovascular disease. Echocardiography was performed before and at peak CPET. Cut-off values for stress-induced left and right ventricular diastolic dysfunction (LVDD/ RVDD) were E/e’>15; E/e’>6, respectively. Correlation analysis was done between CPET parameters and stress E/e’. A p-value <0.05 was considered significant.

**Results:**

64% of the patients had stress-induced LVDD; 78% had stress-induced RVDD. Both groups with stress LVDD and RVDD achieved lower load, lower V’O_2_ and O_2_-pulse, besides showing reduced ventilatory efficiency (higher VE/VCO_2_ slopes). None of the CPET parameters were correlated to stress-induced left or right E/e’.

**Conclusion:**

There is a high prevalence of stress-induced diastolic dysfunction in non-severe COPD patients with exertional dyspnea, free of overt cardiovascular disease. None of the CPET parameters correlates to stress-induced E/e’. This demands the performance of Exercise stress echocardiography (ESE) and CPET for the early detection and proper management of masked HFpEF in this population. (Arq Bras Cardiol. 2021; 116(2):259-265)

## Introduction

Cardiovascular abnormalities are common in chronic obstructive pulmonary disease (COPD).^1,2^ Arterial stiffness is present even in mild COPD patients free of cardiovascular diseases. It is an independent cardiovascular risk factor that contributes for the development of diastolic dysfunction.^3^ Dyspnea and exercise intolerance are common symptoms for both COPD and diastolic dysfunction.^4^ Recent studies with large patient cohorts have identified a cardiovascular phenotype in COPD patients who present with a different clinical course and prognosis.^5^ Early diagnosis and management is, therefore, very important from the clinical point of view.

Cardiopulmonary exercise testing (CPET) may distinguish cardiac and respiratory dyspnea or diminished physical activity.^6-9^ The combination of exercise stress-echocardiography (ESE) and CPET is a reliable approach to identify patients with masked heart failure with preserved ejection fraction (HFpEF). Moreover, the results from invasive measurements are comparable to data obtained with non-invasive studies during ESE.^10^

The aims of our study were: 1) to detect the frequency of subclinical left ventricular (LV) and right ventricular (RV) diastolic dysfunction in non-severe COPD patients free of cardiovascular disease; 2) to establish a correlation between cardiopulmonary exercise and echocardiographic parameters for diastolic dysfunction (E/e^’^).

## Materials and Methods

### Patients and Study Protocol

This was a retrospective study conducted with 224 clinically stable outpatients diagnosed with COPD at the University Hospital for Respiratory Diseases “St. Sophia”, Sofia. Only 163 of them met the inclusion criteria for non-severe COPD – forced expiratory volume in the first second higher than 50% (FEV_1_>50%). All subjects had exertional dyspnea, but a total of 104 patients (64 men, 40 women; mean age of 62.9±7.5 years) were considered eligible, assuming the exclusion criteria. The recruitment period was between May 2017 and April 2018, and was approved by the local Ethics Committee (protocol 5/12.03.2018). All participants signed the informed consent form before the study initiation.

The following exclusion criteria were considered: 1) left ventricular ejection fraction (LVEF) <50%; 2) left ventricular diastolic dysfunction at rest higher than first grade; 3) echocardiographic findings suggesting pulmonary hypertension (systolic pulmonary arterial pressure >36 mmHg, tricuspid regurgitation (TR) jet maximum velocity >2.8 m/s; 4) valvular heart disease; 5) documented cardiomyopathy; 6) severe uncontrolled hypertension (systolic blood pressure >180 mmHg and diastolic blood pressure >90 mmHg); 7) atrial fibrillation or malignant ventricular arrhythmia; 8) ischemic heart disease; 9) anemia; 10) diabetes mellitus; 11) cancer; 12) chronic kidney disease (CKD); 13) recent chest or abdominal surgery; 14) recent exacerbation (over the last three months); 15) recent change (over the last three months) in medical therapy.

## Procedures

### Pulmonary Function Testing

All subjects underwent preliminary clinical examination including chest X-ray, spirometry, electrocardiography, echocardiography. Patients eligible for the study performed spirometry and an exercise stress test. They were performed on Vyntus, Cardiopulmonary exercise testing (Carefusion, Germany), in accordance with the European Respiratory Society (ERS) guidelines.^11^Only patients with mild to moderate airway obstruction (FEV1 >50%) were selected.

### Stress Test Protocol – Cardiopulmonary exercise testing (CPET)

A continuous ramp protocol was applied according to guidelines.^6^ After two minutes of unloaded pedaling (rest phase 0W), a three-minute warm-up phase (20W) followed. The test phase included 20W/2min load increments. Patients were instructed to pedal with 60 rotations per minute.

Expiratory gases were collected on a breath-by-breath basis. Peak VO_2_ was expressed as the highest 30-second average value, obtained during the last stage of the exercise test. The peak values of VO_2_ are expressed as -O_2_ ml/kg/min. Ventilatory efficiency (VE/VCO_2_) was measured by the V-slope method. Peak respiratory exchange ratio (pRER) was the highest 30-second value averaged in the last stage of the test. RER >1.10 at the end of the ESE-CPET test was considered as achievement of maximal effort.

### Echocardiographic Methods

M-mode, two-dimensional and Doppler echocardiography were performed.^12,13^ Apical four-chamber views were used to measure chamber volumes based on Simpson’s modified rule and LV ejection fraction was considered preserved if >50%. Doppler tissue echocardiography (DTE) analysis was performed in the septal-lateral dimension of mitral annulus, and lateral dimension of tricuspid annulus to assess myocardial systolic (S) and diastolic (E’, A’) waves of LV and RV. The E’ value was used as average of medial and lateral measurements. Peak E/e’ ratio >15 was considered a marker for stress-induced left ventricular diastolic dysfunction.

Right ventricular systolic function was assessed using tricuspid annular plane systolic excursion (TAPSE) and tissue Doppler S peak velocity. Right ventricular wall thickness (RVWT) was measured from the subcostal long-axis view at the tip of the anterior tricuspid leaflet in end-diastole. Pulmonary pressure was calculated directly by sampling the tricuspid insufficiency and indirectly by the acceleration time (AT) on pulmonary flow. Right atrium volume index (RAVI) was measured with the right ventricular end-systolic volume by the Simpson’s modified rule. Stress-induced RV diastolic dysfunction was considered if stress-induced E/e’ ratio >6. All parameters were measured at end-expiration and in triplicate during different heart cycles.^14^

### Statistical Analysis

Demographic and clinical data were presented with descriptive statistics. The Kolmogorov-Smirnov test was used to explore the normality of distribution. Continuous variables in each group of subjects were expressed as median and interquartile range when data was not normally distributed and as mean ± standard deviation (SD) if normal distribution was observed. Categorical variables were presented as proportions. Data were compared between patients with and without LVDD, as well as between patients with and without RVDD. The unpaired Student’s t test was used to analyze normally distributed continuous variables. The Mann-Whitney-U test was used in other cases. Categorical variables were compared by the χ^2^ test or the Fisher’s exact test. The Spearman’s rank correlation was used to assess the association between CPET parameters and stress-induced E/e’ ratio for the left and right ventricles.

In all cases, a p value of less than 0.05 was considered significant, as determined with SPSS® 13.0 Software (SPSS, Inc, Chicago, Ill) statistics.

## Results

In the COPD group, 30% (32/104) of patients had grade I LVDD at rest; 14% (15/104) had grade I RVDD at rest and only 3% (4/104) had both RV and LVDD at rest. After CPET, the stress-echocardiography established that 64% (67/104) of the subjects had stress-induced LVDD, and 78% (82/104) had stress-induced RVDD. All patients with stress-induced LVDD also had stress-induced RVDD. The demographic and clinical data of patients are listed in Table 1. The echocardiographic parameters of patients are shown in Table 2. Except for RAVI, RVWT, acceleration time and systolic pulmonary arterial pressure after load, no other significant differences were found between patients with and without LVDD (Tables 1 and 2). The results for patients with and without RVDD were similar (Tables 1 and 2).

**Table 1:** – Anthropometric and cardiopulmonary characteristics of patients with and without LVDD and RVDD

	Patients w/o stress LVDD (37)	Patients with stress LVDD (67)	Patients w/o stress RVDD (22)	Patients with stress RVDD (82)
**Demographic data**				
Age, year	60.44 ± 7.72	64.16 ± 6.97*	6.95±7.36	63.74±7.60*
Male:Female gender, n	21:16	44:23^ǂ^	14:8	50:32^ǂ^
Packet, years	27.21 (23.87-31.76)	33.79 (30.51± 37.87)^†^	26.52 (23.46-30.43)	32.11(28.82-36.13)^ǂ^
Body mass index, kg/m^2^	27.00 (24.75-31.00)	27.96 (22.75-30.75)^†^	28.00 (25.25-30.5)	26.52 (22.72-30.61)^†^
**Respiratory function **				
FVC, l/min	2.06 (1.76-3.09)	2.34 (1.77-3.09)^†^	2.05 (2.11-3.73)	2.21 (1.71-2.93)^†^
FEV_1_, l/min	1.31 (0.94-1.53)	1.36 (1.14-1.75)^†^	1.60 (1.15-2.42)	1.52 (1.14-1.75)^†^
FEV_1_/FVC %	60.5 (46.91-67.47)	53.30 (45.76-66.55)^†^	65.50 (54.81-68.82)	62.59 (46.57-66.79)^†^
**Acid-base balance**				
pO_2_, mmHg	68.60(63.4-71.8)	71.35 (64.7-74)^†^	67.20 (63.56-71.68)	70.6 (63.2-74)^†^
pCO_2_, mmHg	32.30 (30.1-35.37)	37.65 (32.5-40)^†^	34.73 (31.27-39.21)	35.7 (32.5-40)^†^
Sat, %	94.9 (94.4-95.25)	95.00 (94.02-95.67)^†^	94.75 (92.67-95.0)	95.00 (93.9-95.5)^†^
**CPET parameters**				
Peak Load, W	82.75 (69.8-89.1)	76.05 (68.4-92.1)^†^	86.66 (78.65-94.76)	▪73.08 (68.93-83.16)^†^
Peak VE, l/min	40 (34-52.5)	38.50 (32-48)^†^	41.1 (32.12-48.17)	39.07 (31.89-48.32)^†^
Peak V’O_2_, ml/min/kg	14.30(12.6-16.15)	13.90 (12.67-15.7)^†^	14.30 (12.6-16.15)	13.40(15.77-12.55)^†^
RER	1.06 (0.98-1.19)	1.09 (1.00-1.28)^†^	1.05 (0.98-1.18)	1.08 (1.01-1.19)^† ^
Peak O_2_ pulse ml/min/kg	9.80 (9.5-12.2)	7.90 (6.15-9.32)^†^	9.51 (9.02-13.1)	▪7.92(6.27-9.84)^†^
Peak VE/VCO_2_ slope	34.08 (33.98-36.72)	36.93 (34.19-38.74)^†^	34.11 (33.78-36.89)	▪36.98 (34.26-38.91)^†^

*Unpaired t test; †Mann-Whitney U test; ǂchi-square test; §Abbreviations: LVDD: left ventricular diastolic dysfunction; RVDD: right ventricular diastolic dysfunction; O_*2*_ pulse: oxygen pulse; FVC: forced vital capacity; VE: minute ventilation; RER: respiratory exchange ratio; V’O_*2*_: oxygen consumption; VE/VCO_*2*_ slope: ventilatory efficiency; ▪p<0.05 between patients with and without LVDD; ▪▪p<0.05 between patients with and without RVDD.

**Table 2:** – Echocardiographic parameters of patients with and without LVDD and RVDD

	Patients w/o stress LVDD (37)	Patients with stress LVDD (67)	Patients w/o stress RVDD (22)	Patients with stress RVDD (82)
**LV structural parameters **				
LVEF, %, Simpson	63.50(60-66)	60.00(57-65)*	65.00(60-66)	61.00 (67-65)*
Septum, mm	12.00(11-13)	12.00(11-13) *	12.00 (11-12.75)	12.00 (11-13)*
PW, mm	12.00(11.75-12)	12.00(11-13) *	12.00 (11.25-12.75)	12.00 (11-13)*
**LV functional parameters at rest**				
E/A ratio	0.79(0.75-0.85)	0.85 (0.76-1.20)*	0.78 (0.76-0.83)	0.84 (0.75-1.21.)*
E/e’ aver ratio	6.66 (6.25-8.33)	6.97 (5.76-8.15)*	6.96 (6.27-8.33)	6.66 (5.63-8.1)*
**LV functional parameters after exercise stress test**				
E/A ratio	1.25(0.8-1.5)	ǂ1.73 (1.55-2.00)*	1.22 (0.88-1.37)	ǂǂ1.71 (1.5-2.00)*
E/e’ aver	8.07 (6.7-9.6)	ǂ17.33 (15.71-8.46)*	8.12 (7.25-10)	ǂǂ17.14 (14.66-18.39)*
**RV structural parameters **				
RAVI, ml/m^2^	17.57 (16.07-19.97)	ǂ22.66 (21.31-24.13)*	16.55 (15.81-17.54)	ǂǂ22.27 (20.65-23.85)*
RWT, mm	5.00 (4.5-6.5)	ǂ6.50 (6-7)*	5.00 (4.12-5.00)	ǂǂ6.50 (6.00-7.00)*
TAPSE,mm	23.00 (22.00-26.00)	22.00 (21.00-23.00)*	23.00 (21.25-26.00)	22.00 (21-23.5)*
**RV functional parameters at rest**				
E/A ratio	0.83 (0.75-0.95)	0.69 (0.62-0.75)*	0.83 (0.76-1.16)	0.71 (0.66-0.83)*
E/e’ aver	5.47 (4.56-5.69)	4.16(3.33-5.00)*	5.47 (4.56-5.69)	4.54(3.33-5.22)*
AT, msec	170 (163.75-180)	170(160-180)*	170 (165-180)	170(160-180)*
sPAP, mmHg	26.00 (25-28)	28.00 (25-30)*	25.00 (23-27)	28.00 (25-30)*
**RV functional parameters after exercise stress test**				
E/A ratio	1.26 (1.09-1.48)	1.31(1.18-1.49)*	1.28 (1.14-1.5)	1.37 (1.22-1.52)*
E/e’ aver	6.21 (5.38-7.89)	10.83 (9.04-13.23)*	6.92 (5.46-8.00)	ǂǂ11.25 (9.00-13.33)*
AT, msec	165(155-175)	ǂ105(95-110)*	162.5(155-170)	ǂǂ110(95-115)*
sPAP, mmHg	32.00(30-33.25)	ǂ38.00 (36-42)*	32.00 (30-33.75)	ǂǂ 38.00 (35-40)*

*Mann-Whitney U test; LVDD: left ventricular diastolic dysfunction; RVDD: right ventricular diastolic dysfunction; LVEF: left ventricular ejection fraction; RAVI: Right atrium volume index; TAPSE: tricuspid annular plane systolic excursion; PW: posterior wall; SPAP: systolic pulmonary arterial pressure; RWT: right ventricular wall thickness; AT: accelaration time. ǂp<0.05 between patients with and without LVDD; ǂǂp<0.05 between patients with and without RVDD.

Exercise capacity was reduced in COPD patients with stress-induced right and left diastolic dysfunction, compared to those without it (Table 1). The COPD-RVDD/LVDD patients achieved lower load, lower VO_2_ and O_2_-pulse. They performed with significantly higher VE/VCO_2_ slopes (Table 1). None of the CPET parameters was associated with stress-induced left or right E/e’ ratio (Table 3.)

**Table 3:** – Correlation analysis between respiratory and cardiopulmonary exercise testing parameters with stress-induced E/e’ ratio for the left ventricle/right ventricle, respectively

Parameters	LVDD		RVDD	
	Spearman rho	p-value	Spearman rho	p-value
Peak Load , W	0.02	0.84	0.03	0.78
Peak VE, l/min	0.02	0.85	0.12	0.28
PeakVO_2_, ml/min/kg	0.12	0.56	0.03	0.73
RER	0.06	0.74	0.12	0.27
PeakO_2_ pulse ml/min/kg	0.10	0.60	0.11	0.32
Peak VE/VCO_2_ slope	0.35	0.07	0.02	0.80
FVC, l/min	0.28	0.11	0.10	0.34
FEV_1_, l/min	0.01	0.95	0.04	0.71

LVDD: left ventricular diastolic dysfunction; RVDD: right ventricular diastolic dysfunction; RER: respiratory exchange ratio; VO_*2:*_ oxygen consumption; VE/VCO_*2*_ slope: ventilatory efficiency; FVC: forced vital capacity; VE: minute ventilation.

## Discussion

Our main findings were: 1) 64% of the patients with non-severe COPD and exertional dyspnea who are free of clinically evident cardiovascular disease have stress-induced LVDD; 1) 78% of the same group of patients have stress-induced RVDD; 3) none of the CPET parameters was correlated with stress-induced E/e’ ratio either in the left or the right ventricle. To our knowledge, this is the first study using combined ESE-CPET in non-severe COPD patients with exertional dyspnea and free of overt cardiovascular diseases. Stress-induced increase of E/e’ ratio >15 of the left ventricle was detected in 64% of them; stress-induced elevation of E/e’ ratio >6 of the right ventricle was met in 78% of cases. We cannot compare our data to other studies of non-severe COPD patient populations because most of them report on the incidence of diastolic dysfunction at rest+^15-17^

Nedeljkovic et al. performed ESE in a population of 87 hypertensive patients with exertional dyspnea and normal left ventricular function. They found in 9.2% of the patients a stress ratio E/e’ >15.^18^ Kaiser et al. also investigated a general population of 87 patients with exertional dyspnea and reported diastolic dysfunction in 9% of them.^19^

The higher prevalence of stress diastolic dysfunction that we describe in COPD patients confirms that COPD itself is a cardiovascular risk factor.^20,21^Arterial stiffness is a feature of COPD, regardless of the smoking burden. The ventricular wall stress seen during respiration is also reported as an independent pathophysiological mechanism for LV remodeling in mild COPD patients without overt cardiovascular pathology.^22^ Both arterial stiffness and ventricular wall stress cause diffuse LV fibrosis in COPD patients free of cardiovascular diseases.^23,24^

In our study, patients with stress-induced diastolic dysfunction (both LVDD and RVDD) achieve lower load, VO_2_ and O_2_-pulse and perform with significantly higher VE/VCO_2_ slopes. None of the CPET parameters, however, correlates with stress E/e’ ratio (neither in LV nor RV). These findings are similar to what have been reported in the general population. Nedeljkovic et al. detected lower load, lower oxygen consumption and lower ventilatory efficiency in hypertensive patients with exertional dyspnea and stress-induced LVDD.^18^ Kaiser et al. described increased heart rate reserve and reduced oxygen pulse in a general population of patients with exertional dyspnea.^19^ Guazzi et al. also established an association between diastolic dysfunction (E/e’ ratio) and peak oxygen consumption, ventilatory efficiency and heart rate recovery.^25^ In Guazzi’s group of patients with overt cardiovascular pathology and normal echocardiography at rest, ventilatory efficiency correlated best to peak E/e’ ratio>15. The clinical advantage of VE/VCO_2_ ratio as the best predictor of stress E/e’ ratio was also confirmed in the diastolic heart failure patients analyzed by Nedeljkovic et al.^18^ Kaiser et al. do not support such conclusions, emphasizing the importance of the increased heart rate reserve and diminished oxygen pulse as predictors of stress E/e’ ratio in the general population of patients with exertional dyspnea and free of overt cardiovascular disease.^19^

It seems that the CPET parameters may help in the differential diagnosis of dyspnea in the general population, as well as in patients with diagnosed cardiovascular pathology.^6-9^ According to our results, in COPD patients, these are not reliable clinical parameters that may serve as independent predictors for cardiovascular abnormality and, thus, are not applicable in the diagnostic algorithm of masked diastolic dysfunction.

Our findings support the presence of functional impairment in non-severe COPD patients with exertional dyspnea and free of overt cardiovascular disease. The performance of tissue Doppler imaging during exercise demonstrates the complex heart-lung interaction and the effort-induced changes, which increase cardiac functional impairments that may not be evident at rest. Our findings support the current recommendations for ESE-CPET as a tool for early detection of HFpEF.^6^As none of the cardiopulmonary exercise testing parameters proved to be predictive of stress-induced LVDD/RVDD, stress echocardiography has great clinical relevance for an accurate diagnosis of cardiac and respiratory pathology in non-severe COPD patients with exertional dyspnea.

### Study limitations

The study had the following limitations: 1) relatively small sample size; 2) lack of body plethysmography and diffusion capacity measurement, which are informative for the proper assessment of dyspnea; 3) COPD patients experience enhanced pressure swings during the respiratory cycle, and the measurement was performed at the end of expiration, which may influence results; 4) measurements were made in early recovery period (approximately 2 min) after symptom-limited exercise. The timeline of changes of pulmonary and intrathoracic pressures during the brief interval between peak exercise and their measurement in early recovery is not well known and it could, therefore, be underestimated.

## Conclusion

There is a high prevalence of stress-induced diastolic dysfunction in non-severe COPD patients with exertional dyspnea, free of overt cardiovascular disease. None of the CPET parameters correlates with the stress-induced E/e’ ratio. Therefore, the performance of ESE-CPET is needed for the early detection and proper management of masked HFpEF in this population.
